# Measuring exercise in eating disorder patients: a Delphi study to aggregate clinical and research knowledge

**DOI:** 10.1186/s40337-022-00641-7

**Published:** 2022-09-12

**Authors:** Astrid Harris, Phillip Aouad, Melissa Noetel, Phillipa Hay, Stephen Touyz

**Affiliations:** 1 The University of Sydney, Sydney, Australia; 2 Western Sydney University, Sydney, Australia

**Keywords:** Eating disorders, Exercise, Measurement, Questionnaire

## Abstract

**Background:**

Exercise is a prominent feature of most eating disorders, and has been shown to have a number of detrimental effects on treatment outcome. There is some disagreement in the literature regarding the construct of compulsive exercise, and assessment and treatment varies significantly. This study therefore aimed to aggregate expert clinicians’ and researchers’ views on how to define and measure compulsive exercise in eating disorder patients. The expert panel was also asked about questionnaire design, and possible problems when measuring compulsive exercise.

**Method:**

This study used the Delphi method to establish consensus amongst an expert panel. Three successive rounds of questionnaires were distributed to the panel over a period of six months. The first round consisted of four open-ended questions regarding the definition and measurement of compulsive exercise in eating disorder patients. For Round 2, 70 statements were derived from the answers, and panelists were asked to rate each item on a Likert-based scale. An 85% consensus level was chosen. In Round 3, 44 statements were re-rated by the panel.

**Results:**

Seventeen of 24 participants completed all three rounds of the study. Consensus was achieved for 63% of the items, while 18.5% reached near consensus, and 18.5% did not reach consensus after Round 3. The panel agreed on a number of important aspects of compulsive exercise. Several suggestions regarding the format of a questionnaire assessing this behavior were also endorsed. The panel further identified common difficulties when assessing compulsive exercise in eating disorder patients, notably a lack of consensus still apparent in the literature.

**Conclusion:**

The current findings constitute a further step towards a unified definition of compulsive exercise, and contribute important suggestions to the measurement of this behavior.

## Plain English summary

Exercise is an important feature of most eating disorders. Researchers have found that people with an eating disorder exercise because they feel driven to do so, or because it helps them cope with negative emotions. This type of exercise is called ‘compulsive exercise’, and it can have detrimental physical and mental effects. There is some disagreement in the literature about how to define compulsive exercise, and there are many ways to measure and treat it. The aim of this study is to collect expert researchers’ and clinicians’ views of compulsive exercise, and elicit suggestions regarding how to reliably measure this behaviour in order to promote consensus in the field.

## Background

Compulsive exercise behaviour has been observed in people with eating disorders since earliest clinical descriptions, and there are well established links to adverse outcomes such as longer hospitalisation, higher risk of and earlier relapse, treatment dropout, suicidality, and higher risk of a chronic outcome in eating disorder patients who exercise compulsively [1–6]. Compulsive exercise is also clearly linked to eating disorder pathology, indicated by more severe eating disorder symptoms and related features such as drive for thinness, body dissatisfaction, and perfectionism in compulsive exercisers [7, 8].

Despite the clear relevance of compulsive exercise to eating disorder pathology and treatment success, varied conceptual approaches to exercise in eating disorders have made it difficult to arrive at a widely accepted definition of this eating disorder symptom [9, 10]. While a recent Delphi study has shown significant progress regarding the definition of compulsive exercise, the results apply to a subgroup of eating disorder patients, namely adolescents with anorexia nervosa [10]. In addition, the study was focused on intervention and on difficulties when treating adolescents with anorexia nervosa who also engage in compulsive exercise behaviour [10]. In contrast, the present Delphi study focuses on a transdiagnostic definition of compulsive exercise in individuals of all ages, and on designing a valid and reliable instrument to measure this behaviour.

There is significant variety in the assessment and treatment of exercise in eating disorders [11]. Researchers and clinicians alike have long disagreed on fundamental aspects, such as whether qualitative or quantitative elements are more important when assessing exercise behaviours in eating disorders [9]. Findings emerging in the last decades have brought some clarification regarding a definition of compulsive exercise. It now seems clear that compulsive exercise is a multidimensional construct, and that there is a qualitative difference between the exercise behaviour of eating disorder patients and healthy individuals [12]. Research has also shown that there are compulsive elements to exercise in people with eating disorders, and that amount of exercise alone is not predictive of compulsivity or eating pathology [13, 14]. The term ‘compulsive exercise’ has also been endorsed as the preferred term for researchers and clinicians in a recent Delphi study [10]. Further aspects of compulsive exercise that were endorsed by research include affect regulation, perfectionism, and guilt, while weight and shape concerns also remain an important part of compulsive exercise [12].

Dittmer and colleagues [15] have recently proposed a clinical definition of compulsive exercise behaviour in line with the different aspects identified by research. Criterion A highlights the highly driven and rigid nature of compulsive exercise and its function in regulating emotions, criterion B addresses functional aspects such as the impact of compulsive exercise on an individual’s time, daily routine and occupational and social functioning, and criterion C requires the individual to recognize their behaviour as unreasonable. This definition encompasses many of the abovementioned aspects of compulsive exercise. The current study presents an opportunity to lend credibility to their definition by comparing it to an expert panel’s view of compulsive exercise.

Due to the number of different definitions of exercise behaviour, various instruments can be found in the literature. Examples include the Commitment to Exercise Scale [16], the Obligatory Exercise Questionnaire [17], the Reasons for Exercise Inventory [18], the Exercise Addiction Inventory [19] and the Exercise Dependence Scale [20]. These instruments vary considerably in their conceptual underpinnings. For example, the Exercise Addiction Inventory [19] leans heavily on addiction theory, the Exercise Dependence Scale [20] uses the DSM criteria for substance abuse, and the Obligatory Exercise Questionnaire [21] relies in part on quantitative aspects of compulsive exercise behaviour, which has been shown to be inadequate to distinguish between compulsive and healthy exercise [22]. While these instruments have been used with individuals with eating disorders in the absence of measures specific to this group, research has clearly shown qualitative differences between exercise behaviour of individuals with versus without an eating disorder, such as exercise to control weight and shape[7, 23], level of compulsivity [24], and exercise rigidity [25], components not specifically addressed in the questionnaires mentioned above. These findings clearly highlight a need for measures designed for this particular group [12, 26, 27].

There are two instruments specific to compulsive exercise in eating disorders, the Exercise and Eating Disorders questionnaire [27], and the Compulsive Exercise Test [26, 28]. These instruments differ from the ones mentioned above in that they were developed specifically for the assessment of exercise in eating disorders, considering research in the area and clinical expertise. They hence take into account eating disorder specific aspects that were not considered in the development of other questionnaires assessing exercise behaviour.

Both the Compulsive Exercise Test and the Exercise and Eating Disorders questionnaire have good psychometric properties, and are a promising start to the quest for reliable assessment tools [28]. However, they have a number of shortcomings. For example, different studies have found different factor structures for the Compulsive Exercise Test, and the Mood Improvement subscale has not been consistently related to eating disorder pathology [23, 29–33]. The Exercise and Eating Disorders has so far only been validated in small inpatient samples [28]. Thus further research into theoretical models of compulsive exercise is needed to facilitate improvements to definition and measurement [28].

Indeed, a recent study investigating the validity of the Compulsive Exercise Test in a large clinical sample suggests that theoretical models of compulsive exercise may need to be improved after finding that the total score of the CET likely does not measure the same construct in different eating disorder diagnoses [34]. Furthermore, additional factors such as trait compulsivity and interpersonal difficulties have recently been shown to play a role in compulsive exercise behaviour [35]. Other research has suggested that current models of compulsive exercise insufficiently address its function, do not discriminate between exercise in anorexia nervosa and other eating disorders, and fail to consider the influence of cultural attitudes towards body shape [36]. These findings suggest that defining and assessing compulsive exercise in eating disorders is an ever-evolving process. An expert panel’s opinion could hence contribute significantly to clarifying and advancing current theoretical models and measurement approaches.

A widespread understanding of the nature of compulsive exercise would likely facilitate comparable research, and assist in improving existing assessment tools. The primary aim of the current Delphi study is therefore to gain a clearer understanding of how expert eating disorder clinicians and researchers define compulsive exercise, which underlying constructs they believe to be important, how a questionnaire measuring compulsive exercise should be structured, and what common difficulties are when measuring compulsive exercise.

## Methods

### Study design

The present study employed the Delphi technique, which has been widely used in various areas of health research, including eating disorders, to achieve consensus in a field [10, 37–40]. The Delphi technique involves a multi-stage process with participants who are considered experts on a given topic. It is particularly useful when there is limited research on a topic, or a lack of agreement and evidence in the literature [41]. Due to the variety of approaches to the definition and measurement of exercise in eating disorders, the Delphi technique seems appropriate to advance knowledge in this field.

### Participants and recruitment

Purposive sampling was used to identify potential participants worldwide. Potential participants were identified through an online search, and invited via email to take part in the study. The online search focused on key publications in the field of exercise in eating disorders, and on identifying individuals with a high level of clinical expertise and knowledge in eating disorders. Inclusion criteria were defined a priori. To be included in the study, potential participants were required to meet at least one of the following criteria:

(I) be the primary author on least one peer-reviewed publication, or authored a book or book chapter in the field of exercise in eating disorders, and/ or
(II) have been engaged in clinical practice with eating disorder patients for at least 3 years.

Based on these criteria, a list of 66 potential participants was created. The literature offers no clear recommendations regarding Delphi panel size [41]. While a panel size of roughly 20 members is considered appropriate, there are studies in the mental health literature with much smaller panel sizes [41]. Therefore, current study aimed to recruit a sample size of 20 to 25 participants to try and obtain a diverse, yet representative sample. An invitation email describing the study was sent to the individuals identified as potential panel members by the online search. Participants were informed that the study would consist of three rounds of questionnaires, and were encouraged to only participate if they could commit to complete all three rounds. Ethical clearance was granted by the University of Sydney Ethics Committee (2020/681).

### Procedure

**Fig. 1:**
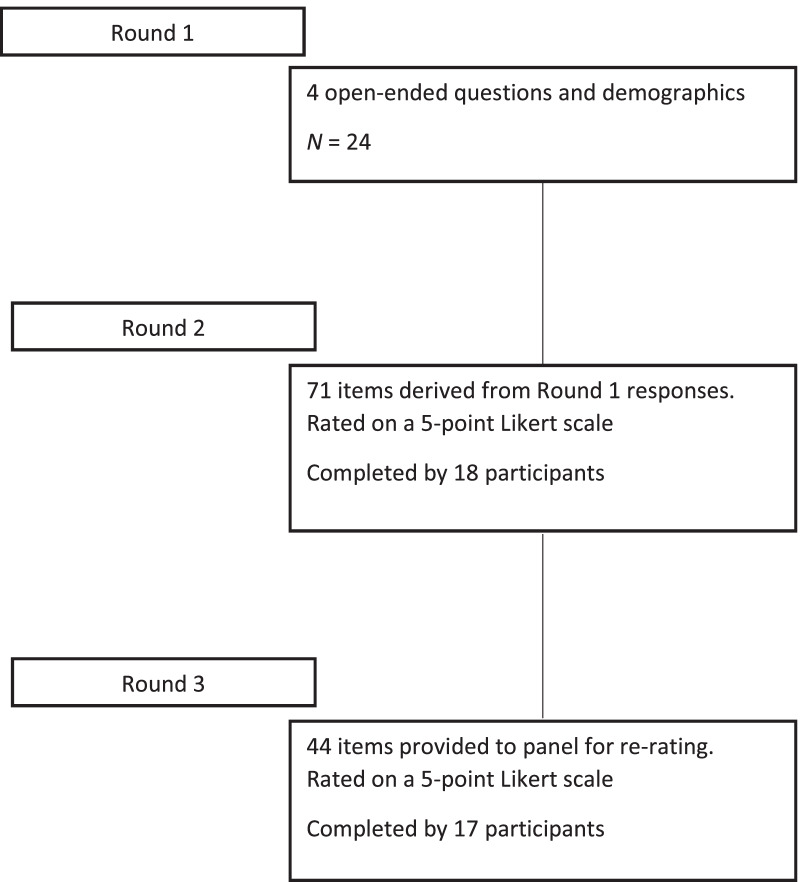
Flow diagram of response process

Three successive rounds of questionnaires were distributed to the panel via email. Figure 1 details participant responses and number of questions for each round. Each email included a link to the survey, using the online software program Qualtrics. Surveys were distributed synchronously and over a period of six months between April and October 2021. Participants were sent a reminder email if they hadn’t completed the survey three weeks after distribution to reduce attrition. Participants remained anonymous to other members of the panel throughout the study, but were given the option to be acknowledged in the publication.

### Defining consensus

An established definition of consensus is lacking in the literature, and consensus levels tend to vary between studies [41]. For the purposes of this study, a conservative consensus level of 85% was defined prior to commencing the study, a decision also made by various other authors in the eating disorders field [10, 39]. An item was deemed to have achieved consensus if it was rated ‘strongly agree’ or ‘somewhat agree’ to indicate agreement, and ‘strongly disagree’ or ‘somewhat disagree’ to indicate disagreement by > 85% of participants. Near consensus was reached when between 75 and 85% of participants chose ‘strongly (dis)agree or ‘somewhat (dis)agree’. No consensus was achieved when < 75% of the panel indicated agreement or disagreement.

#### Round 1

The first round of questionnaires consisted of a number of demographic items to verify that inclusion criteria were met, and four open-ended questions exploring the definition and assessment of compulsive exercise in eating disorder patients. Despite the many terms used to describe exercise behaviours in eating disorder patients, evidence suggests that the term compulsive exercise’ is the most accurate [9, 14]. Hence this term was used in all three rounds of questionnaires.

The four Round 1 questions were:

1. What words would you use to define compulsive exercise in eating disorder patients?
2. Compulsive exercise is conceptualised across a range of constructs. Which concepts/ constructs do you consider essential when assessing the compulsivity of exercise in eating disorder patients?
3. What should be included in a measure of exercise behaviours for eating disorder patients? E.g. what should the question format be, number of questions, any other comments you may have about the structure of such a measure?
4. What are the challenges when measuring compulsive exercise, or exercise in general, in eating disorder patients?

#### Round 2

Seventy-one statements were created from responses to Round 1 using content analysis, and re-submitted to the panel for rating. Participants were asked to rate each of the statements on a 5-point Likert scale ranging from ‘*strongly disagree’* to ‘*strongly agree’*. Questions that reached consensus in Round 2 were excluded from Round 3.

#### Round 3

Questions which did not reach the required consensus level in Round 2 were re-submitted to the panel. Each individual panel member was presented with their response from Round 2 and the overall group response in form of a histogram. This allowed participants to consider their response in light of the overall group response. Participants were then asked to consider whether they wanted to change their response. A comment box was added to the end of Round 3 giving participants the opportunity to comment on the overall study and individual items, and address any additional points they considered important.

### Data analysis

Content analysis was used to analyse Round 1 data. Content analysis is a qualitative research method that is used to identify categories and themes within a set of data [42]. The goal is to reliably condense a large amount of text into a summary of key points, while correctly interpreting meaning [43, 44].

Answers were categorised into common themes for each of the four open-ended questions in Round 1 by two of the authors. Similar responses were merged. To increase reliability, statements were created using participants’ own words where possible. A high level of agreement was reached between the researchers, further increasing reliability. Quantitative results from Round 2 and Round 3 were analysed using IBM SPSS Statistics for Windows software version 27. Frequency scores were calculated for each item to establish whether consensus had been achieved.

## Results

### Response rate

Sixty-six potential participants who met our criteria for inclusion were identified by the online search. Twenty-four individuals chose to participate in Round 1. Three participants declined, and 41 participants did not respond to the invitation. Eighteen participants completed Round 2, and 17 participants completed Round 3. Two participants completed Round 1 but did not meet inclusion criteria, and did therefore not participate in Rounds 2 and 3. Their response was not considered for Round 1 data analysis. Two participants were from the same research group and submitted a joint answer to Round 1. They were subsequently asked to complete Rounds 2 and 3 separately.

### Panel characteristics

Of the 17 participants who completed all three rounds, 15 were female and two were male. Participants ranged between 28 and 62 years of age. Panelists were located in different locations globally. Countries include Australia, Canada, Germany, Norway, Sweden, the United States and the United Kingdom. Panelists had specialised in the area of exercise in eating disorders for between 5 and 40 years. Sixteen panel members had at least one peer-reviewed publication in the field, and 16 had the required clinical experience in the treatment of eating disorders. 15 participants met both inclusion criteria, and two participants met one of the inclusion criteria. Thirteen participants (76.47%) were psychologists, one a psychiatrist, one a dietician and exercise physiologist, one an exercise physiologist and one a physiotherapist (5.88% respectively). Please see Table 1 for panel characteristics. Of the seven participants who dropped out after Round 1, one was a researcher, two were psychiatrists, and four were psychologists. Participants came from various different locations such as the United States, the United Kingdom, Sweden, and Australia.

**Table 1:** Baseline characteristics of study panellists (*N* = 17)

*Profession*	[*n*(%)]
Psychologist	13 (76.47)
Psychiatrist	1 (5.88)
Dietician and exercise physiologist	1 (5.88)
Exercise physiologist	1 (5.88)
Physiotherapist	1 (5.88)
*Geographical location of predominant workplace*	
Australia	2 (11.76)
Canada	1 (5.88)
Germany	2 (11.76)
Norway	3 (17.64)
Sweden	3 (17.64)
United Kingdom	2 (11.76)
United States	5 (29.41)
Currently engaged in clinical practice with eating disorder patients	Yes: 11 (64.70) No: 6 (35.5)
Ever engaged in clinical practice with eating disorder patients	Yes: 16 (94.12) No: 1 (5.88)
Published in the area of exercise in eating disorders	Yes: 16 (94.12) No:1 (5.88)

	[*M*(SD)]
Hours focused on clinical work with individuals with an eating disorder	26 (20.17)
Years worked clinically with eating disorder patients	10.59 (8.66)

### Overall results

In Round 2, 28 of the 71 questions reached consensus, and 17 reached near consensus. In Round 3, a further 17 questions reached consensus at the 85% level, 13 questions reached near consensus. In total, 45 out of 71 statements reached consensus (62.86%), and 13 reached near consensus (18.57%), leaving 13 statements (18.57%) that did not reach the required levels.

### Definition of compulsive exercise in eating disorder patients

Fifteen statements were generated from participants’ responses to this question in Round 1. Of the 15 statements, ten statements (62.50%) reached consensus and two reached near consensus (12.5%). Three items did not reach consensus (18.75%). A list of items that did not reach consensus can be found in Table 6.

*Question 2*: Construct of compulsive exercise

Seventeen statements were generated from responses to this question in Round 1. The highest amount of disagreement between panel members occurred in this section. Twelve statements (70.59%) reached consensus, and five statements (29.41%) did not reach consensus. There were no near consensus statements.

*Question 3*: Design of a questionnaire measuring compulsive exercise

Sixteen statements were generated for this question from Round 1. Eight statements reached consensus (50%) and five statements reached near consensus (31.25). Three items did not reach consensus (18.75%).

*Question 4*: Difficulties measuring compulsive exercise

Twenty three questions were generated from responses to this question. Thirteen questions reached consensus (56.52%). Six questions reached near consensus (26.09%), and four items did not reach consensus (17.39%).

## Discussion

A total of 62.86% of statements reached consensus, representing an equal or higher amount of agreement amongst the expert panel compared to other Delphi studies in the field [38, 45].

### Definition of compulsive exercise

The panel endorsed a number of aspects that characterise compulsive exercise in individuals with eating disorders. It was agreed that compulsive exercise is a complex and multifaceted symptom which is common in eating disorders. Compulsive exercise was viewed as a means to compensate for calorie intake, which was not considered to be adequate in individuals engaging in compulsive exercise. This may point to compulsive exercise being viewed as predominantly a symptom of restrictive Anorexia Nervosa, given that Bulimia Nervosa patients are generally not underweight [46, 47]. A lack of satisfaction with the amount of exercise, a significant impact on an individual’s daily routine and functioning, and proneness to injury were also endorsed.

The panel disagreed on whether compulsive exercise can be an enjoyable experience, and whether individuals who engage in compulsive exercise behaviour realise that their behaviour is pathological. This raises a definitional issue, as compulsions are defined in the DSM-V as ‘repetitive behaviours that the person feels driven to perform’ that are ‘aimed at preventing or reducing distress’ (APA, 2013). This definition clearly views compulsive behaviour as a strategy to manage negative affect, and gives rise to the question whether something that is classified as compulsive can also be enjoyable. A lack of support for exercise to increase positive affect in eating disorder patients also supports the DSM-V definition [48–50].

No consensus was reached on whether individuals who engage in compulsive exercise have difficulty noticing bodily signals such as pain, hunger, thirst or exhaustion. Individuals with eating disorders typically continue to exercise even when they are ill or injured [15]. Clarifying whether this behaviour is due to not noticing physical symptoms, or simply ignoring them, could benefit interventions for compulsive exercise behaviour (Table 2).

**Table 2:** Definition of compulsive exercise

Item	*M*	SD	Mode	% of panellists showing agreement	Consensus achieved
Compulsive exercise is common in individuals who have an eating disorder	4.61	0.50	5	100	Yes (Round 2)
Compulsive exercise is a complex and multifaceted eating disorder symptom	4.00	0.87	4	94	Yes (Round 3)
Compulsive exercise is the result of a negative reinforcement process where an emotion perceived as aversive is alleviated by engaging in the behaviour	3.89	0.83	4	88	Yes (Round 2)
Compulsive exercise is a means to compensate for energy intake	4.33	0.67	4	89	Yes (Round 2)
Individuals with an eating disorder can have obsessive thoughts about exercise without engaging in compulsive exercise behaviours	4.24	0.75	4	94	Yes (Round 3)
When unable to engage in compulsive exercise, individuals often experience distress or feelings such as guilt, anger or shame	4.72	0.58	5	94	Yes (Round 2)
People who exercise compulsively are rarely satisfied with the amount or intensity of physical activity they engage in	4.24	0.83	5	76	Near (Round 3)
Compulsive exercise impairs an individual’s daily routine, occupational functioning and/or social relationships	4.33	0.84	5	89	Yes (Round 2)
Individuals with an eating disorder tend to prioritise compulsive exercise over other activities	4.24	0.44	4	100	Yes (Round 3)
Compulsive exercise can be seen as a maladaptive coping mechanism	4.50	0.51	4	100	Yes (Round 2)
Individuals who engage in compulsive exercise are more prone to injury	4.47	0.87	5	88	Yes (Round 3)
Individuals with an eating disorder who engage in compulsive exercise generally do not consume enough calories to nourish themselves adequately	3.94	0.75	4	82	Near (Round 3)

### Construct of compulsive exercise

The lowest agreement was reached on statements pertaining to relevant aspects of the construct of compulsive exercise. This was expected given the extent of disagreement in the literature about how to conceptualise this behaviour. The findings highlight that there is still uncertainty about the role of certain factors in the construct of compulsive exercise, such as exercise to increase positive affect. Research has confirmed that exercising to improve affect is not necessarily exclusive to eating disorder patients, and may hence be considered an adaptive rather than a pathological exercise motive [48, 49, 51].

Interestingly, the panel was divided on the importance of qualitative versus quantitative factors in compulsive exercise behaviour. This is somewhat surprising, given that the literature clearly points to a higher importance of qualitative aspects, and amount or frequency of exercise have not been found to be linked to eating pathology in both eating disorder patients and healthy individuals [9, 14, 25]. However, recent findings illustrate that quantitative aspects may play a role in distinguishing exercise behaviours of different eating disorder subgroups. Some recent studies found a difference between exercise obsessions and exercise compulsions in eating disorder patients, where even individuals who do not engage in high levels of compulsive physical activity score high on measures of compulsive exercise, indicating the presence of obsessive thoughts about exercise [32, 52]. This indicates that it may be useful to include quantity of exercise behaviours in a construct of compulsive exercise to be able to distinguish between these subgroups. This information could also lead to a differentiation in treatment approach depending on whether the primary problem is obsessive thoughts or compulsive behaviour, and may help clinicians manage physical complications that arise from engaging in compulsive exercise behaviour.

The panel agreed that motivation to exercise and the function of exercise behaviour is important when considering the exercise in healthy individuals versus exercise behaviour in eating disorder patients, further suggesting that there is a qualitative difference between the two groups. This is in line with numerous studies that found exercise to regulate negative affect to be unique to eating disorder patients when compared to healthy controls [53–55].

The panel also agreed on a number of aspects of the construct of compulsive exercise that are consistent with the current literature in the field. For example, negative affect regulation, weight and shape concerns, rigidity, perfectionism and a compulsive drive to exercise were all endorsed as part of compulsive exercise behaviour [13, 24, 53, 54, 56–60]. This further supports the notion that compulsive exercise is a multidimensional construct, as suggested by previous studies [12] (Table 3).

**Table 3:** Construct of compulsive exercise

Item	*M*	SD	Mode	% of panellists showing agreement	Consensus achieved
Regulation of negative affect such as guilt, anxiety or sadness is a primary function of compulsive exercise in eating disorder patients	4.06	0.24	4	100	Yes (Round 3)
Eating disorder patients exercise to alleviate negative affect	4.33	0.59	4	94	Yes (Round 2)
Weight and shape concerns are a primary motivator for compulsive exercise behaviours in eating disorder patients	4.11	0.90	4	89	Yes (Round 2)
Rigid exercise routines and an inability to allow flexibility in one’s exercise schedule are characteristic of compulsive exercise in eating disorder patients	4.72	0.46	5	100	Yes (Round 2)
Individuals who engage in compulsive exercise tend to continue to exercise despite injuries or illness	4.50	0.62	5	94	Yes (Round 2)
Compulsive exercise is closely linked to eating disorder pathology	4.50	0.62	5	94	Yes (Round 2)
Compulsive exercise behaviours tend to take up significant amounts of time	4.24	0.44	4	100	Yes (Round 3)
Perfectionism is a part of compulsive exercise behaviour	3.82	0.53	4	76	Near (Round 3)
A perceived internal drive to exercise is an important part of the construct of compulsive exercise	4.00	0.61	4	94	Yes (Round 3)
It is important to assess an individual’s motivation to engage in compulsive exercise	4.72	0.46	5	100	Yes (Round 2)
Obsessive thoughts about exercise and/or compulsive exercise behaviours are main features of the way eating disorder patients exercise	4.12	0.33	4	100	Yes (Round 3)
Individuals who engage in compulsive exercise tend to be overly preoccupied with their exercise schedule and performance	3.94	0.66	4	88	Yes (Round 3)
Demographic data such as physical condition, BMI, age, gender, and energy intake are important to include in an assessment of compulsive exercise	4.41	1.06	5	88	Yes (Round 3)
A measure of compulsive exercise should assess for beliefs about exercise	4.56	0.78	5	94	Yes (Round 2)
It is possible to distinguish between compulsive and healthy exercise by looking at the function of exercise for the individual	3.82	1.02	4	76	Near (Round 3)

### Questionnaire format

Regarding the format of a questionnaire assessing compulsive exercise behaviour, the panel agreed that the measure should include all relevant aspects of compulsive exercise behaviour as identified above in order to provide a comprehensive assessment. It was however suggested that the measure be kept as brief as possible so it can be integrated into routine assessment of eating disorders. A possible solution that emerged from Round 1 responses proposes to include a screening questionnaire, followed by a more extensive questionnaire or interview if indicated. It appears that a balance between a comprehensive enough assessment and a practical and time-saving assessment is needed. Given the complex and multifaceted nature of compulsive exercise in eating disorders, a more thorough assessment may be warranted for individuals who obtain high scores on measures of compulsive exercise. This appears particularly important given that compulsive exercise often precedes the onset of an eating disorder, and can persist after eating disorder treatment has been completed [61].

The panel also agreed that the measure should include a number of items assessing frequency of exercise in addition to qualitative aspects. Given that there was disagreement pertaining to the relative importance of qualitative versus quantitative aspects of compulsive exercise, it is not surprising that the panel advocated for the inclusion of some frequency items. Given that recent research has shown that frequency of exercise behaviours may be useful to distinguish between exercise obsessions without a behavioural component, and exercise compulsions, frequency items may be beneficial to gain more information about how compulsive exercise manifests in different eating disorder subgroups [32, 52, 62].

An interesting point emerged regarding incidental exercise. The panel suggested that it would be beneficial to assess for all types and levels of physical activity, not just those typically considered exercise such as running or weightlifting. Particularly anorexia nervosa patients tend to exhibit behaviours such as clenching muscles, walking for long periods of time, or standing instead of sitting [15]. It may be important to account for these differences in an assessment of compulsive exercise, particularly as findings show that individuals with anorexia nervosa tend to underreport how much incidental physical activity such as walking they engage in [15, 63] (Table 4).

**Table 4:** Questionnaire design

Item	*M*	SD	Mode	% of panellists showing agreement	Consensus achieved
A questionnaire assessing compulsive exercise should be long enough to be comprehensive, so that multiple components of the construct can be assessed adequately	4.33	0.97	5	89	Yes (Round 2)
A thorough assessment needs to include both questionnaires and a semi-structured interview	4.24	5	1.20	76	Near (Round 3)
A questionnaire assessing compulsive exercise should assess for all types and levels of elevated physical activity, even those not typically considered formal exercise such as walking, standing or clenching muscles	4.61	5	0.78	94	Yes (Round 2)
Answers should be in the form of a Likert scale	4.18	5	0.81	76	Near (Round 3)
When asking about specific exercise behaviours, the questionnaire should differentiate between physical activity at work/school, active transportation, household work, and sport and leisure time to get an accurate idea of the amount of physical activity	4.18	4	0.64	88	Yes (Round 3)
It is preferable to have a short screening questionnaire, followed by a longer questionnaire or interview if indicated	4.41	5	0.80	82	Near (Round 3)
The measure should reflect the multidimensional nature of compulsive exercise, and should cover the domains that research has identified as relevant, such as affect regulation, compulsivity, guilt, rigidity, and weight and shape concerns	4.89	5	0.32	100	Yes (Round 2)
The measure should assess whether individuals continue to exercise despite illness, injury or other adverse indications	4.56	5	0.71	89	Yes (Round 2)
Q42 The questionnaire should include some questions about amount (frequency, intensity and duration of exercise behaviours) as well as type of exercise	4.82	5	0.53	94	Yes (Round 3)
A questionnaire assessing compulsive exercise should be as short as possible so it can be integrated into routine assessment of eating disorders	3.71	4	0.92	76	Near (Round 3)
A measure of compulsive exercise behaviours needs to capture psychosocial impairment caused by this behaviour	4.39	4	0.51	100	Yes (Round 2)
It is important to assess the history of compulsive exercise behaviours	4.18	4	0.73	82	Near (Round 3)
It is important to track any changes in exercise behaviour and cognitions about exercise over the course of treatment	4.72	5	0.75	89	Yes (Round 2)

*Question 4*: Difficulties when assessing compulsive exercise behaviour

The panel agreed that the lack of consensus among researchers and clinicians on how to define and measure compulsive exercise has significantly impacted progress in the field, as research is often not comparable. The resulting lack of an international gold standard in the assessment and measurement of compulsive exercise in eating disorders further complicates the establishment of best practice guidelines. Experts clearly view promoting a universally accepted definition of compulsive exercise, creating valid and reliable measures, and establishing treatment guidelines as essential to advancing the field.

Despite not being explicitly asked about, there were a number of comments by the expert panel addressing treatment of compulsive exercise. A lack of exercise professionals in ED treatment, lack of adequate facilities in treatment centres, and not prioritising a return to healthful exercise in treatment were mentioned. Research on the treatment of compulsive exercise has significantly advanced in the last decade. There are multiple treatment approaches specifically for compulsive exercise in eating disorder patients, including the Loughborough Eating Disorders Activity program (LEAP), the Freiburg Sport Therapy Program, and Physical Exercise and Dietary Therapy (Mathisen et al., 2018, Hay et al., 2018, Schlegel et al., 2015). The expert panel endorsed the importance of assigning healthful exercise a more central part in eating disorder treatment, suggesting that these new exercise interventions, while promising, may not yet have permeated general eating disorder treatment.

One of the difficulties pointed out by the panel was that existing measures of compulsive exercise may not be accurate in athletes. Professional athletes exercise–often by necessity–in a way that could be considered unhealthy in normal individuals [64]. For example, athletes often adhere to a strict training schedule, exercise when they are unwell, or make up for missed exercise sessions, behaviour not necessarily pathological in this population. Consequently, compulsive exercise is thought to differ qualitatively between athletes and non-athletes [65]. It is hence questionable whether existing measures are adequate for athletes, and, more importantly, whether they are able to reliably detect compulsive exercise behaviour in athletes with an eating disorder. This problem has been highlighted in the literature, and measures have been developed specifically for athletes [64]. However, a modified version of the Compulsive Exercise Test, the CET-A, has been shown to successfully distinguish between athletes with versus without an eating disorder, indicating that the differences in exercise behaviour between individuals with versus without an eating disorder also apply to this group [66] (Table 5).

**Table 5:** Difficulties when measuring compulsive exercise

Item	*M*	SD	Mode	% of panellists showing agreement	Consensus achieved
Existing measures of compulsive exercise may not be accurate in athletes	4.33	0.97	5	89	Yes (Round 2)
There is a lack of consensus among researchers, clinicians and individuals with eating disorders on how to define and conceptualise compulsive exercise	4.76	0.44	5	100	Yes (Round 3)
The lack of consensus has impacted progress in the field, as research is often not comparable	4.61	0.78	5	89	Yes (Round 2)
The lack of consensus has impacted progress in the field, as research is often not comparable	4.47	0.62	5	94	Yes (Round 3)
Existing measures do not take into account that individuals with an eating disorder may have obsessive thoughts or unhealthy attitudes towards exercise without a behavioural component	4.00	0.71	4	88	Yes (Round 3)
As exercise is a socially accepted behaviour, it may be difficult for eating disorder patients to realise that the way they exercise is pathological in the context of their eating disorder	3.89	1.13	5	89	Yes (Round 2)
A measure assessing compulsive exercise contributes – in some cases unnecessarily- to the already extensive assessment process for individuals with eating disorders	4.89	0.32	5	89	Yes (Round 2, disagree)
Eating disorder treatment should include facilities to teach patients to exercise in a healthful way through in vivo exposure	4.82	0.93	5	100	Yes (Round 3)
Teaching eating disorder patients how to exercise in a healthful way needs to be given more focus in treatment	4.39	0.98	5	94	Yes (Round 2)
It is difficult to measure compulsive exercise accurately due to inaccuracy of self-report	3.82	1.13	4	76	Near (Round 3)
It is difficult to measure compulsive exercise accurately due to varying definitions of what constitutes exercise	4.39	0.50	4	89	Yes (Round 2)
It is difficult to measure compulsive exercise accurately due to patients with eating disorders engaging in a variety of physical activity they may not see as exercise, such as tensing muscles or standing for long periods of time	3.50	1.15	4	94	Yes (Round 2)
It is difficult to measure compulsive exercise accurately due to desirability reporting	3.88	0.86	4	82	Near (Round 3)
It is difficult to measure compulsive exercise accurately due to current questionnaires not assessing for incidental exercise	3.76	0.83	4	76	Near (Round 3)
The accuracy of self-report is likely diminished due to concerns about consequences of answering truthfully	4.29	0.99	5	88	Yes (Round 3)
The accuracy of self-report is likely diminished due to recall bias	3.59	0.94	4	76	Near (Round 3)
The accuracy of self-report is likely diminished due to desirability reporting	3.76	1.09	4	82	Near (Round 3)
There is no international gold standard describing how to best measure and assess compulsive exercise	4.44	0.98	5	94	Yes (Round 2)

Ecological momentary assessment is another, arguably more accurate way to measure quantitative aspects of exercise which also allows researchers to investigate the relationships between exercise behaviour and emotional states [67, 68]. Physical activity is generally measured by an electronic device for multiple days. The panel disagreed on whether this method should be used as an additional assessment tool, and on whether the extensive commitment required by the individual raises ethical concerns. While ecological momentary assessment may not be a feasible tool for a routine assessment, it is certainly useful in investigating the association between emotional states before and after engaging in compulsive exercise [67] (Table 6).

**Table 6:** Items that did not reach consensus

Item	M	SD	Mode	% of panellists showing agreement after Round 3
*Definition of compulsive exercise*				
Individuals who engage in compulsive exercise generally do not find the experience enjoyable	3.24	0.90	3	41 (agree)
Individuals who engage in compulsive exercise have difficulty noticing bodily signals such as pain, hunger, thirst, or exhaustion	3.71	0.77	4	65 (agree)
Individuals with an eating disorder who engage in compulsive exercise tend to realise at some point that their behaviour is excessive	3.12	0.70	3	18 (agree)
*Construct of compulsive exercise*				
Eating disorder patients exercise to increase positive affect	3.47	1.07	4	71 (agree)
Qualitative aspects are more important in the assessment of compulsive exercise than quantitative aspects	3.76	0.97	4	65 (agree)
Questionnaire design				
The questionnaire should include open-ended questions when assessing motivation to exercise and impact of exercise behaviours on life	3.65	1.00	4	59 (agree)
Ecological momentary assessment should be included in the assessment an additional measuring tool	2.94	0.83	3	18 (disagree)
The measure should be able to differentiate between exercise cognitions and behaviours of different eating disorder subtypes	3.47	1.07	3	29 (agree)
*Difficulties*				
Existing measures of compulsive exercise focus too much on cardiovascular exercise and neglect other forms of exercise such as body building/ muscular fitness	3.82	0.95	3	59 (agree)
It can be difficult to distinguish between compulsive exercise and healthy exercise behaviours in individuals with an eating disorder	3.59	1.06	4	71 (agree)
Accelerometry may not be a feasible tool due to ethical concerns such as the extensive commitment required by the individual	2.82	1.07	4	35 (disagree)
It is difficult to measure compulsive exercise accurately due to a lack of reliable measures assessing quantitative aspects	3.41	1.12	4	65 (agree)
The accuracy of self-report is likely diminished due to fear of being judged, shamed or stigmatized	3.41	0.94	4	65 (agree)

### Strengths

The present study addresses a significant gap in the literature on exercise in eating disorders. Results highlight a high level of agreement on a number of important aspects regarding the definition, construct and measurement of compulsive exercise. The study also corroborates previous research showing that compulsive exercise is a multidimensional construct, and that its assessment and treatment are important in the wider eating disorder context. Given the extent of disagreement in the compulsive exercise literature, these results point the way towards a more unified understanding of compulsive exercise.

Dittmer and colleagues’ [15] definition of compulsive exercise endorses regulating adverse emotions as a major reason for engaging in compulsive exercise, highlights the rigid and driven nature of compulsive exercise, and links perfectionism to compulsive exercise behaviour. Other aspects include the impact of compulsive exercise on social and occupational functioning, whether exercise is continued despite injury or illness, and whether enjoyment is derived from exercise. The results of this study lend credibility to their definition, indicating that research may be on its way towards a more unified understanding of compulsive exercise.

Participants’ predominant workplaces were evenly distributed, with ten participants working in English speaking countries, and eight participants working in non-English speaking countries (one participant worked in two countries). This ensures an even representation of various cultural and linguistic backgrounds, and corresponding perspectives on the topic. However, all participants were recruited from countries with a modern Western culture, hence no suggestions can be made regarding possible differences between their approach and the approach of researchers and clinicians from a different cultural background.

### Limitations

The majority of participants were psychologists. Given that clinical guidelines clearly recommend a multidisciplinary approach to assessing and treating eating disorders, more participants from different backgrounds may have been beneficial [69]. However, the panel did include a physiotherapist, a psychiatrist and two exercise physiologists, ensuring that the perspectives of the different groups of professionals typically involved in the assessment and treatment of exercise in eating disorders were included.

Seventeen participants completed all three Delphi rounds. This represents a significant reduction in panel size, despite several strategies to avoid attrition such as a clear explanation of structure and length of the study process, email reminders, and limiting the study to three rounds a priori. However, other relevant studies in the field had significantly larger attrition rates: 40% in Hart & Wade [40], 79.6% in McMaster and colleagues [45], and 32.1% in Strand et al. [70], and comparable Delphi studies had a similar number of participants [71]. Given that there is no clear recommendation for Delphi panel size, and other Delphi studies in the field had a similar number of participants, the panel size is deemed adequate for the purposes of this study.

Two participants from the same research group submitted a common answer for Round 1. This represents a slight deviation from Delphi study protocol. However, the two participants indicated that they had a high level of agreement on the topic, and had written a number of peer-reviewed articles on exercise in eating disorders. It is therefore unlikely that their answers would have differed significantly. The two participants subsequently completed Rounds 2 and 3 separately.

### Implications for future research

The expert panel endorsed a clear need for a universal working definition of compulsive exercise, and for valid and reliable measures to advance assessment. While there are many instruments assessing exercise behaviour, there are currently only two relatively new measures targeted specifically at eating disorder patients [26, 27]. Both of these measures show good psychometric properties, and have been investigated in numerous studies [28]. However, there are some shortcomings that need to be addressed in line with current research. For example, this study and previous studies illustrate that exercise to improve mood is not unique to eating disorder patients, and may therefore not be a relevant part of the assessment [31, 32].

Previous research has largely used instruments that were not designed for exercise in eating disorder patients, leading to the possible exclusion of factors unique to this subgroup. Further research is needed to validate a clinical definition of compulsive exercise along the lines of Dittmer and colleagues [15] to judge which measures are appropriate. Clearly, there is still a need for conceptual research in the field, as well as further development of existing questionnaires to ensure congruence between a definition and model of compulsive exercise, and measures for this behaviour.

While the expert panel did not endorse the use of ecological momentary assessment as a regular tool, using it in research could significantly advance the field. Ecological momentary assessment allows for the investigation of emotional states immediately prior to and after the behaviour, rather than relying on retrospective account [51, 67]. Further research using ecological momentary assessment could therefore pinpoint exactly when emotion regulation happens in relation to compulsive exercise, and further investigate the role of both positive and negative emotionality. This may be particularly useful as there are findings indicating that exercise for mood improvement may be an adaptive response found in exercisers with and without eating disorders rather than a pathological response exclusive to individuals with eating disorders [31, 32]. Clarifying the role of emotion regulation could significantly improve existing models of compulsive exercise, particularly as the panel disagreed on the importance of mood improvement.

To the authors’ knowledge, there currently aren’t any studies specifically targeting eating disorder patients’ perspectives on compulsive exercise. Insight into lived experience could be an important additional perspective to learn more about a complex and multifaceted eating disorder symptom. Other studies in the eating disorders field have recognized the value of lived experience when trying to advance an under-investigated area of eating disorder research (Broomfield, Rhodes & Touyz, 2021). Including the views of eating disorder patients with compulsive exercise behaviour could add important impulses to a clinical definition.

## Conclusion

The panel agreed on a number of important aspects of compulsive exercise such as negative affect regulation, weight and shape concerns, rigidity, perfectionism and a compulsive drive to exercise, lending credibility to both the cognitive behavioural model of compulsive exercise, and Dittmer and colleagues’ definition [15]. The importance of agreeing on such a definition, and the need for reliable and valid instruments was further highlighted by the panel. Addressing shortcomings of existing instruments, and incorporating newly emerging aspects of compulsive exercise could therefore further advance consensus amongst researchers and clinicians, and ultimately benefit eating disorder patients through improved assessment and treatment options. As research on the various aspects of compulsive exercise is evolving continuously, it may be beneficial to approach a definition of compulsive exercise as a work in progress which can be modified considering newly emerging aspects. In more practical terms, given the important role compulsive exercise plays in eating disorder course and recovery, adding a standardised assessment as well as treatment recommendations to best practice guidelines could be helpful to increase successful treatment.

## Data Availability

The datasets analysed during the current study are available from the corresponding author on reasonable request.
